# Cmss1 limits FMDV infection by enhancing antigen presentation and CD8^+^ T cell responses

**DOI:** 10.1128/jvi.01249-25

**Published:** 2025-11-24

**Authors:** Yang Wang, Lihong Zhang, Jieru Deng, Linlin Zheng, Zhihua Chen, Zhao Zhang, Han Zhang, Jingjing Pei, Haixue Zheng

**Affiliations:** 1 State Key Laboratory for Animal Disease Control and Prevention, College of Veterinary Medicine, Lanzhou University, Lanzhou Veterinary Research Institute, Chinese Academy of Agricultural Sciences 12426, Lanzhou, China; 2 Gansu Province Research Center for Basic Disciplines of Pathogen Biology, Lanzhou, China; 3 Department of Microbiology and Immunology, The University of Melbourne, at The Peter Doherty Institute for Infection and Immunity 2281, Melbourne, Victoria, Australia; St Jude Children's Research Hospital, Memphis, Tennessee, USA

**Keywords:** FMDV, single-cell RNA sequencing, dendritic cells, Cmss1, antigen presentation

## Abstract

The foot and mouth disease virus (FMDV) poses a heavy burden on the global swine industry, underscoring the urgent need for long-term protective vaccines to control FMDV infection. Dendritic cells (DCs) are critical in activating innate immune cells and initiating adaptive immune responses. However, the role of DCs in FMDV infection remains poorly understood. In this study, we developed a mouse model lacking the type I interferon receptor for infection, followed by single-cell RNA sequencing to generate a high-resolution map of DCs in the spleens of FMDV-infected mice. Next, we established an evaluation system to investigate the antigen-presenting capacities of gene-edited DCs and mice. The results reveal that FMDV infection alters the proportions of DC subsets and significantly suppresses antigen processing and presentation. Additionally, we identified Cmss1 as a novel host factor that antagonizes the inhibition of the antigen-presenting process caused by FMDV infection, thereby limiting FMDV pathogenicity in mice. These findings provide valuable insights into potential antiviral strategies against FMDV infection.

**IMPORTANCE:**

In this study, we first developed C57BL/6 mice lacking the type I interferon receptor as an infectious model, which was subjected to single-cell RNA sequencing analysis of DC features in response to FMDV infection. We identified VP41_9-30_ as an immunodominant CD8^+^ T cell epitope of FMDV and established an *in vitro* system to evaluate the antigen-presenting capacities. Based on these findings, we validate Cmss1 as a novel host factor in antigen processing and presentation during FMDV infection. Since DCs play critical roles in mediating immune responses, our findings comprehensively characterize the immune features of dendritic cells for the first time and present a new mechanism through which the host defends against FMDV infection, suggesting Cmss1 as a novel potential target for antiviral therapies.

## INTRODUCTION

Foot and mouth disease (FMD) is a widespread disease that affects cloven-hoofed ruminants such as swine, sheep, and cattle. Since livestock is a predominant source of economic income in many countries and regions, the disease significantly burdens the global economy and food security. FMD virus (FMDV), also known as the aphthous fever virus, is a non-enveloped RNA virus belonging to the Picornaviridae family. Seven serotypes of FMDV (A, O, C, SAT1, SAT2, SAT3, and Asia1) exhibit diverse antigenicity. This diversity complicates cross-protection among serotypes, meaning each serotype requires a specific vaccine to provide adequate immunity (1–4). Among all seven serotypes, type O is the most common and poses a significant challenge worldwide. It is responsible for approximately 70% of FMD outbreaks worldwide and is, therefore, considered the most serious FMD serotype (5–7). Upon FMDV infection, the host’s humoral immune system rapidly produces specific antibodies targeting the virus. This antibody-mediated response is essential for neutralizing the virus and can provide protective immunity for months (8). However, recent studies have reported that antibody responses alone cannot entirely prevent FMDV infection (9). Although antibodies are essential for disease defense, they respond relatively late and often do not provide timely protection against infections, while cellular immune responses can elicit an immediate and effective reaction to viral infections. Therefore, clarifying the interaction between FMDV and host cellular immune responses will guide and assist in developing antiviral strategies based on cellular immunity (9, 10). Furthermore, inactivated vaccines that induce antibody-mediated immune protection are widely used in clinical settings; however, latent infections and reinfections among immune animals are quite common, underscoring the limitations of currently available vaccines. Our limited understanding of the T-cell immune mechanisms triggered by FMDV infection remains a fundamental obstacle to the defects in current vaccine development (11–13).

Dendritic cells (DCs) play a crucial role in presenting pathogen antigens and activating the adaptive immune response. Typically, after FMDV infection, DCs internalize the virus and process its antigens. These processed antigens are then presented on the surface of DCs, bound to major histocompatibility complex (MHC) molecules, which subsequently trigger the adaptive immune response to eliminate the infection by activating T cells (14). Studies have indicated that DCs comprise two major subsets: classical DCs (cDCs) and plasmacytoid DCs (pDCs), both of which are essential in sensing viral pathogens and inducing long-term antiviral immunity (15–18), while cDCs are known for their pivotal function in stimulating naïve T cells and orchestrating acquired immune responses (19, 20). According to Guilliams et al., cDCs are categorized into two major lineages: cDC1 and cDC2 (21). Specialized DC subsets exhibit diverse ontogeny, phenotypes, and functions, and many viruses disrupt the functions of DCs to suppress the immune response (22, 23). However, the relationship and regulatory mechanisms between FMDV infection and host antigen presentation remain unclear. Therefore, a comprehensive understanding of the biological characteristics of DCs and their antigen presentation function during FMDV infection is essential for investigating the mechanisms of FMDV immunosuppression and immune evasion and will significantly assist in vaccine improvement.

FMDV infection strongly suppresses the type I IFN response (24–27) and causes disease in cloven-hoofed animals, but not in wild-type (WT) mice. Previous studies have used nude mice as models for FMDV infection (28–30), some of which have restored adaptive immunity by the adoptive transfer of immunocompetent cells (31). However, these models are not ideal for studying DCs. In our study, we established a model of FMDV infection using Ifnar knockout (Ifnar^–/–^) mice on the C57BL/6 background, which effectively replicates the clinical symptoms and pathological changes associated with FMD and serves as an effective animal model to investigate the effects of FMDV infection on antigen presentation function. Furthermore, by utilizing single-cell RNA sequencing (scRNA-seq), we conducted a high-resolution analysis of splenocytes from *Ifnar*^–/–^ mice infected with FMDV at 5 days post-infection (dpi). This approach provided a comprehensive transcriptome landscape and enabled the deconvolution of the genomic profiles of DC subsets. Additionally, we mapped the T cell responses during FMDV infection in mouse spleens and established a system for assessing antigen presentation capacity. Concurrently, we identified Cms1 small ribosomal subunit homolog (Cmss1) as a previously unrecognized host factor that mediates DC responses and antigen processing and presentation during FMDV infection. These findings provide new insights into the pathogenesis of FMDV and aid in developing antiviral strategies.

## RESULTS

### A novel mouse model susceptible to FMDV infection

**Fig 1:**
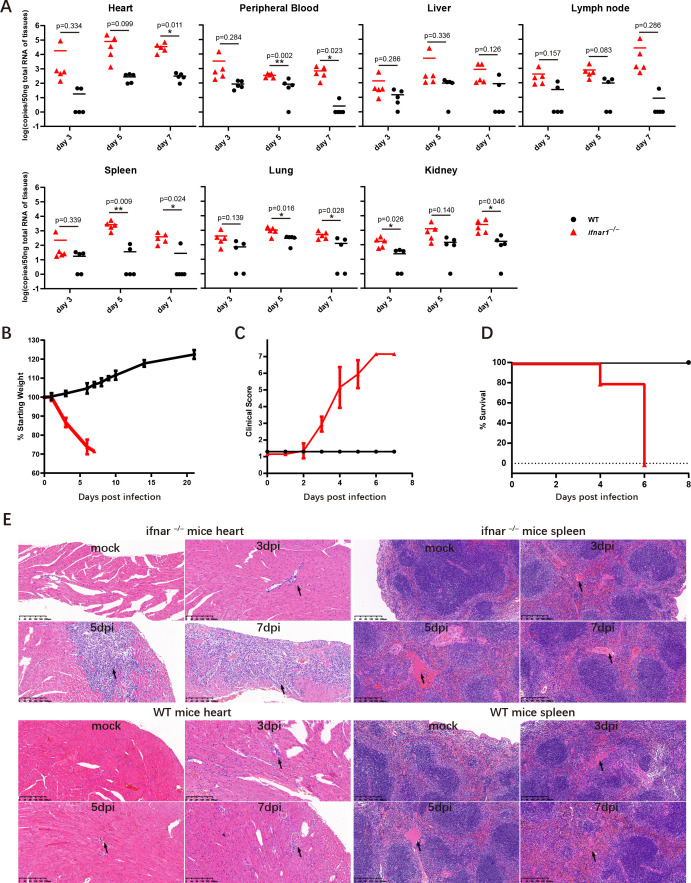
Establishing the *Ifnar^–/–^* mouse model for FMDV infection. WT and *Ifnar*^–/–^ mice at 4 weeks of age were infected with 10^4^ plaque-forming units (PFUs) of FMDV (*n* = 5). Heart, peripheral blood, liver, lymph node, spleen, lung, and kidney were harvested at days 3, 5, and 7 post-infection, and the levels of FMDV were determined using RT-qPCR. (**A**) FMDV at days 3, 5, and 7 post-infection in WT (black circles) and *Ifnar*^–/–^ mice (red triangles) are shown. Five mice were included in each group. (**B–C**) Weight and clinical scores of infected WT (black circles) and *Ifnar*^–/–^ mice (red triangles) were monitored at each time point. (**D**) Survival curves of infected WT (black circles) and *Ifnar*^–/–^ mice (red triangles). (**E**) Representative IHC images of heart and spleen tissues from WT or *Ifnar*^–/–^ mice infected with FMDV at days 3, 5, and 7 post-infection. The images are representative of three independent biological replicates.

To establish a mouse model susceptible to FMDV infection, *Ifnar*^–/–^ and WT C57BL/6 mice were subcutaneously injected with 10^4^ plaque-forming units (PFUs) of FMDV, and the viral RNA loads in the heart, peripheral blood, liver, lymph node, spleen, lung, and kidney were evaluated subsequently. At 3, 5, and 7 dpi, the viral RNA was detectable in all *Ifnar^–/–^* tissues, while viral RNA loads remained extremely low and nearly undetectable in WT mice. It should be noted that viral loads in the tissues of *Ifnar^–/–^* mice were 10 to 10^5^ times higher than those in WT mice, particularly in the spleen (Fig. 1A). Next, we investigated whether *Ifnar*^–/–^ mice showed clinical symptoms after FMDV infection using a clinical criteria scale (Table 1) and weight monitoring. As expected, *Ifnar^–/–^* mice showed significant weight loss and related clinical symptoms (Fig. 1B and C). Notably, 100% of the *Ifnar*^–/–^ mice died from FMDV infection (Fig. 1D). After that, we further determined the pathological effects of FMDV infection. To this end, heart and spleen tissues from *Ifnar*^–/–^ and WT mice infected with FMDV were collected and subjected to clinicopathological analysis. Compared to WT mice, *Ifnar^–/–^* mouse hearts exhibited significantly more severe parenchymatous myocarditis, and their spleens showed more serious acute splenitis, effectively simulating the typical pathological characteristics and clinical symptoms of FMDV infection (Fig. 1E). Finally, flow cytometry was performed to analyze the immune cell composition in the spleen using the gating strategy outlined in Fig. S1A. No significant differences were observed in the proportions of T lymphocytes, B lymphocytes, DCs, and macrophages, indicating that the susceptibility of *Ifnar^–/–^* mice, compared with WT mice, was not due to the depletion of immune cells ( Fig. S1B). This further suggests that *Ifnar^–/–^* mice do not impact the adaptive immune response triggered by FMDV infection. Collectively, these results indicate that *Ifnar^–/–^* mice are a more suitable animal model for *in vivo* infection and immunological studies than WT mice.

**TABLE 1:** Clinical scores on a 7-point scale

Score	Description	Appearance	Mobility	Attitude
1	Healthy	Smooth coat	Active, scurrying, and burrowing	Alert
2	Slightly Ruffled	Slightly ruffled coat	Active, scurrying, and burrowing	Alert
3	Ruffled	Ruffled coat throughout body. A "Wet" appearance.	Active, scurrying, and burrowing	Alert
4	Sick	Very ruffled coat. Slightly closed, inset eyes.	Walking, but no scurrying.	Mildly lethargic
5	Very Sick	Very ruffled coat. Closed, inset eyes.	Slow to no movement.	Extremely lethargic
6	Euthanize	Very ruffled coat. closed, inset eyes. Moribund requiring humane euthanasia.	No movement or uncontrollable, spastic movements.	Completely unaware or in noticeable distress
7	Deceased	/^*a*^	/	/

^*a*^ "/" indicates not applicable.

### The global cell distribution in the mouse spleen infected with FMDV

**Fig 2:**
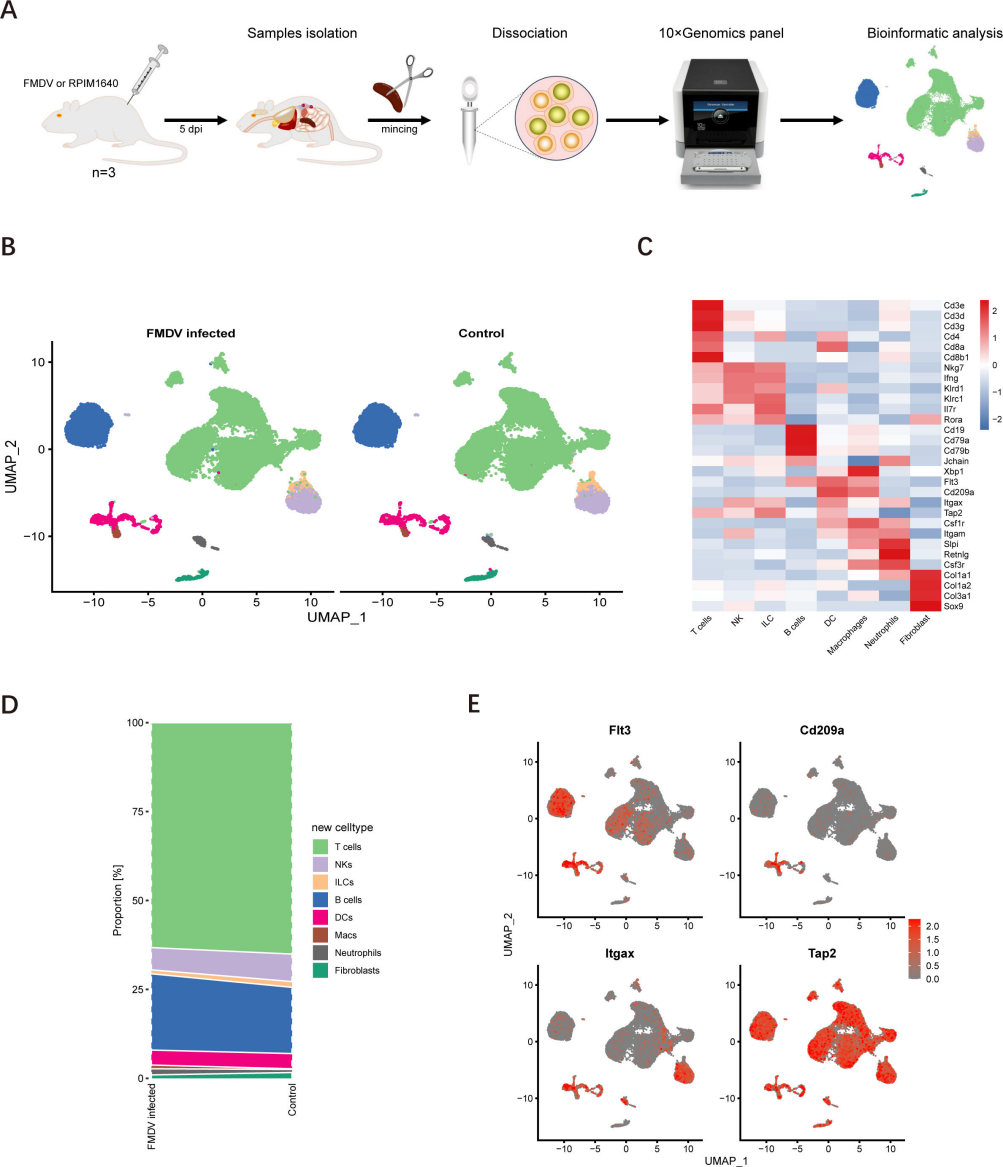
Global distribution of immune cell distribution in mouse spleen during FMDV infection. Single-cell transcriptomic profiling of mouse spleen and verification of immune cell distribution during FMDV infection. (**A**) Schematic of the study. Single cells were isolated from mock- and FMDV-infected mice (*n* = 3) at 5 dpi. All single cells were sequenced using the 10X Genomics facility. (**B**) Cellular maps of immune cells at single-cell resolution level displayed on UMAP dimension reduction according to cell types. (**C**) Heatmaps of the marker genes used for cluster annotations. (**D**) Bar plots of the proportions of different cell types from mock- and FMDV-infected mice. (**E**) UMAP plots colored for gene expression in DCs.

The spleen, one of the peripheral lymphoid organs, plays a critical role in trapping antigen-bearing DCs and initiating adaptive immune responses. Pathogen antigens are transported to the spleen from infection sites, primarily by DCs. Considering the high levels of viral loads (Fig. 1A) and the lesions observed in spleens (Fig. 1E), this organ was selected for further investigation. We chose 5 dpi as the sampling timepoint based on our viral replication assay, which showed that viral loads peak at this stage, indicating active infection. To comprehensively characterize the molecular profile of DCs in the spleens of FMDV-infected mice, scRNA-seq was conducted on freshly collected spleens, capturing a total of 25,379 and 30,440 high-quality cells from FMDV-infected and mock-infected mice, respectively, at 5 dpi (Fig. 2A).

According to the expression of canonical markers, eight significant populations were identified via graph-based clustering of uniform manifold approximation and projection (UMAP) (Fig. 2B and C). These populations included T cells, natural killer cells (NKs), innate lymphoid cells (ILCs), B cells, DCs, macrophages (Macs), neutrophils, and fibroblasts (Fig. 2B through D). Among these cells, the DC populations exhibited high levels of Flt3, CD209a, Itgax, and Tap2 (Fig. 2E), suggesting the accuracy of the identification. Notably, there are no significant changes in the proportion of any cell types, including DCs (Fig. 2B and D). These findings were further validated by flow cytometric analysis (Fig. S2A and B). To further explore the potential shifts in the global proportion of immune cells during secondary FMDV infection, four experimental groups were established with initial infectious doses of 0, 0, 50, and 50 PFUs per mouse (*n* = 5) on day 0, followed by secondary infectious doses of 0, 5,000, 0, and 5,000 PFUs per mouse on day 30, respectively. All spleens were harvested at 35 dpi and subjected to flow cytometric analysis following the gating strategies (Fig. S1A). Consistent with previous results, the distribution of all cell types remained relatively stable at the global level (Fig. S2C). Based on the above results, we comprehensively defined the cellular distribution globally in the spleen of FMDV-infected mice.

### APC subset distribution in the mouse spleen after FMDV infection

**Fig 3:**
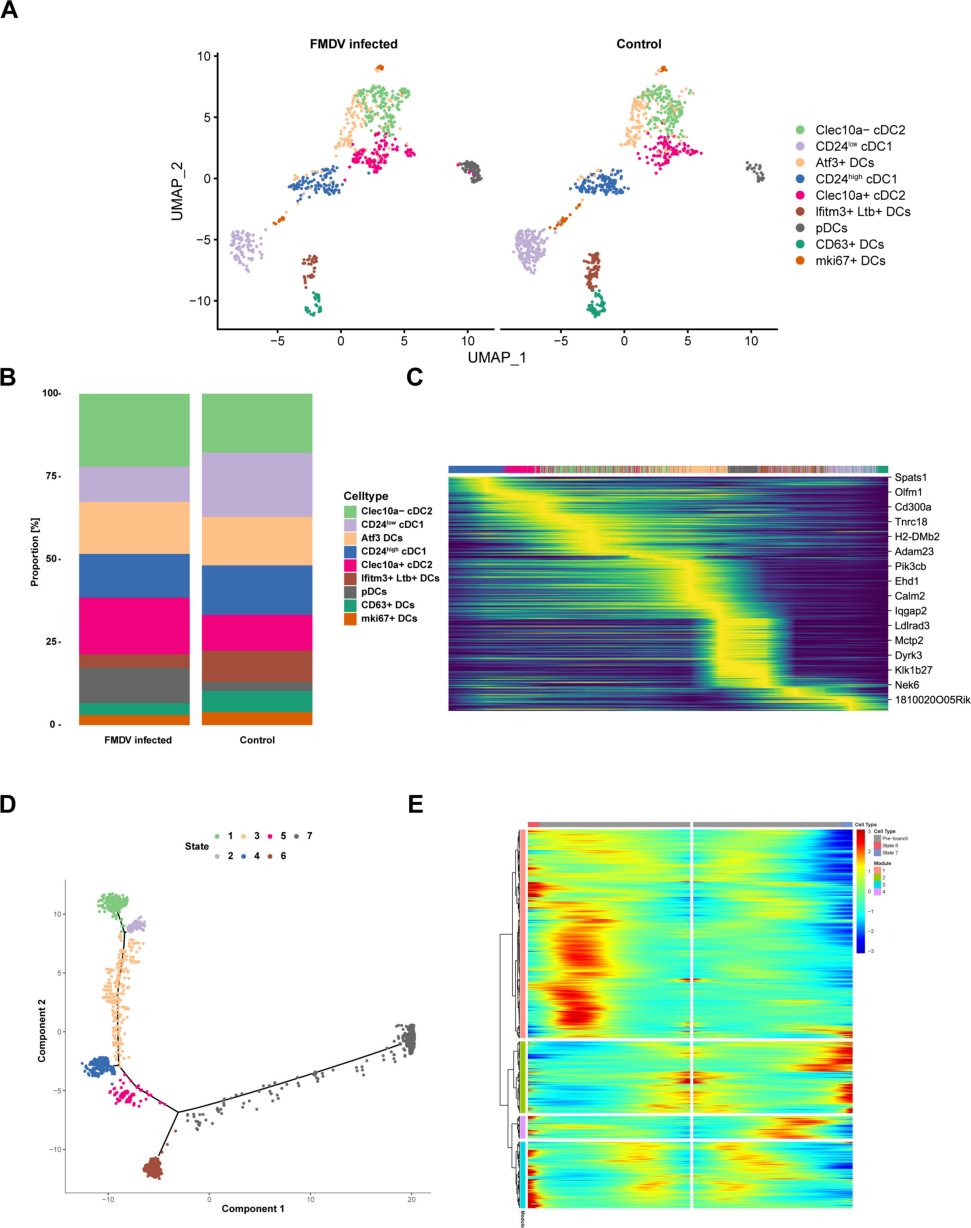
Diversity of DC subsets in FMDV-infected mice spleen. (**A**) The UMAP plots showing DC subsets populations in mouse spleen during FMDV infection. Clusters are annotated according to markers in Fig. S3A. Bar plots show the proportions of different DC subsets from mock- and FMDV-infected mice. (**C**) RNA velocity analysis of spleen scRNA-seq data. (**D**) The pseudotime analysis of DCs in FMDV-infected mice spleen. (**E**) Gene expression dynamics model for DCs based on the pseudotime results.

Since different subsets of DCs play varied roles in pathogen recognition and immune activation, each DC subset may serve as the coordinator for a specific function module. Therefore, we further characterized the distribution of DC subsets in the mouse spleen, and nine subsets were recovered and manually annotated based on signature markers (Fig. 3A; Fig. S3A). They were Clec10a^−^ cDC2, CD24^low^ cDC1, Atf3^+^ DCs, CD24^high^ cDC1, Clec10a^+^ cDC2, Ifitm^+^Ltb^+^ DCs, pDCs, CD63^+^ DCs, and Mki67^+^ DCs. Notably, we observed decreases in DC1, Ifitm^+^Ltb^+^ DCs, and CD63^+^ DCs and increases in DC2 and pDCs in infected samples (Fig. 3B). According to previous research, cDC1s primarily prime CD8^+^ T cells, while cDC2s are crucial for CD4^+^ T cell activation (32). The reduction in cDC1s indicates a potential inhibition of CD8^+^ T cell responses during FMDV infection. Moreover, RNA velocity analysis (33) showed three main directions from the center (CD24^high^ cDC1) to CD24^low^ cDC1, CD63^+^ DCs, and Atf3^+^ DCs (Fig. S3B through D Fig. 3C).

Furthermore, pseudotime analysis was used to dissect the cell states of DC subsets during FMDV infection. We found that DCs were divided into two branches: CD24^low^ cDC1 was significantly enriched at the end of the upper branch, while CD24^high^ cDC1 and DC2s were distributed around the origin. Meanwhile, other DC subsets exhibited varied distributions: pDCs were present at the origin, Atf3^+^ DCs clustered at the end of the lower branch, and Ifitm^+^Ltb^+^ DCs, along with CD63^+^ DCs, exhibited aggregation at the upper tail (Fig. S3E). These six DC subsets were categorized into seven different states (Fig. 3D), with terminal states 6 and 7 showing different function modules (Fig. 3E) and dynamic compositional changes (Fig. S4A). These two states of cells had similar compositions at the starting point (mainly composed of Mki67^+^ DCs, pDCs, CD24^high^ cDC1, and cDC2) and subsequently changed along the pseudotime (cells of state 6 were primarily composed of Atf3^+^ DCs, while state 7 contained four DC subsets, namely, Mki67^+^ DCs, CD63^+^ DCs, Ifitm^+^Ltb^+^ DCs, and CD24^low^ cDC1, at the end) (Fig. S4A). Based on our findings, the numbers of CD63^+^ DCs, Ifitm^+^Ltb^+^ DCs, and CD24^low^ cDC1 remarkably decreased during FMDV infection (Fig. 3B), indicating the reduction of cells of state 7. According to GO analysis, the genes in module 2, which were significantly upregulated in state 7, strongly correlated with ribosome-related functions (Fig. S4B). These findings indicate that ribosome-associated genes could play a crucial role in the interactions between FMDV and the host. Altogether, these data revealed the spectrum of DC lineage, their development, and the molecular profiles of individual DC subsets.

### FMDV infection suppresses MHC expression and reprograms ribosome-related genes in DCs

**Fig 4:**
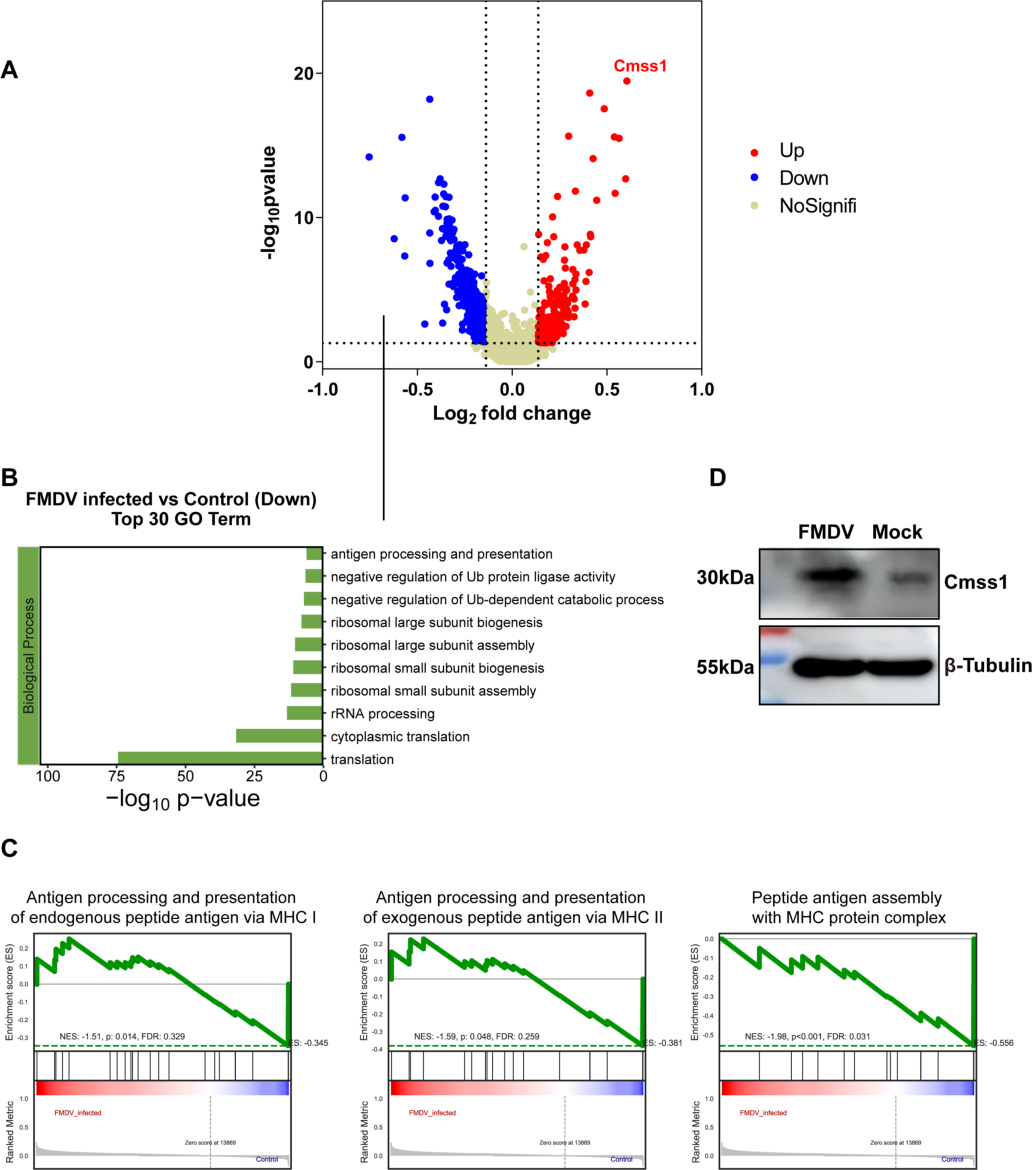
FMDV infection reprograms the gene expression of DCs. Characterization of DC gene expression profiles in the spleens of FMDV-infected mice. (**A**) Volcano plots of DEGs of FMDV- vs mock-infected DCs at 5 dpi. The top one up-regulated DEG is depicted. The horizontal dash line indicates *P* value = 0.05; the vertical dash lines indicate log_2_FC = 0.1 or −0.1. (**B**) Gene Ontology (GO) analysis of genes significantly downregulated during FMDV infection. (**C**) Changes in pathway activities scored in DCs by GSEA, with enriched GO terms. (**D**) Western blot for cmss1 in FMDV- and mock-infected porcine lung epithelial cells. Tubulin was used as loading control.

DCs play a central role in antigen presentation. Our study identified differentially expressed genes (DEGs) in DCs during FMDV infection, revealing 342 downregulated and 234 upregulated genes (Fig. 4A). Among the DEGs, we identified significant downregulation of MHC class I (H2-D1 and H2-K1) and MHC class II (H2-Eb2, H2-Aa, and H2-Ab1) in DCs (Fig. S5A), which is consistent with the known ability of FMDV to suppress MHC class I in swine cells (34). Moreover, Gene Ontology (GO) and Gene Set Enrichment Analysis (GSEA) analyses indicated that the antigen processing and presentation process was downregulated by FMDV infection (Fig. 4B and C). These findings suggest that FMDV actively inhibits antigen processing and presentation in DCs. Notably, the downregulated genes were markedly enriched in ribosome-related biological processes, such as ribosomal large and small subunit assembly and ribosomal biogenesis (Fig. 4B; Fig. S5B). Interestingly, Cmss1, which plays a crucial role in ribosomal biology, was significantly upregulated in response to FMDV infection (Fig. 4A; Fig. S5C). Based on the scRNA-seq results, we further explored the potential regulation of Cmss1 in porcine lung epithelial cells infected with FMDV using Western blot analysis and observed similar results (Fig. 4D). Ribosomes typically act as protein factories and are essential in immune responses. Our findings revealed that FMDV infection results in the disruption of ribosomal function and the inhibition of antigen processing and presentation pathways. However, intriguingly, the expression level of the ribosomal functional protein CMSS1 significantly increased following virus infection. Next, we aim to further investigate whether CMSS1 plays a regulatory role in host antigen presentation, which is crucial for our comprehensive understanding of FMDV immune regulation.

### Screening of immunodominant T cell epitopes derived from FMDV structural proteins

**Fig 5:**
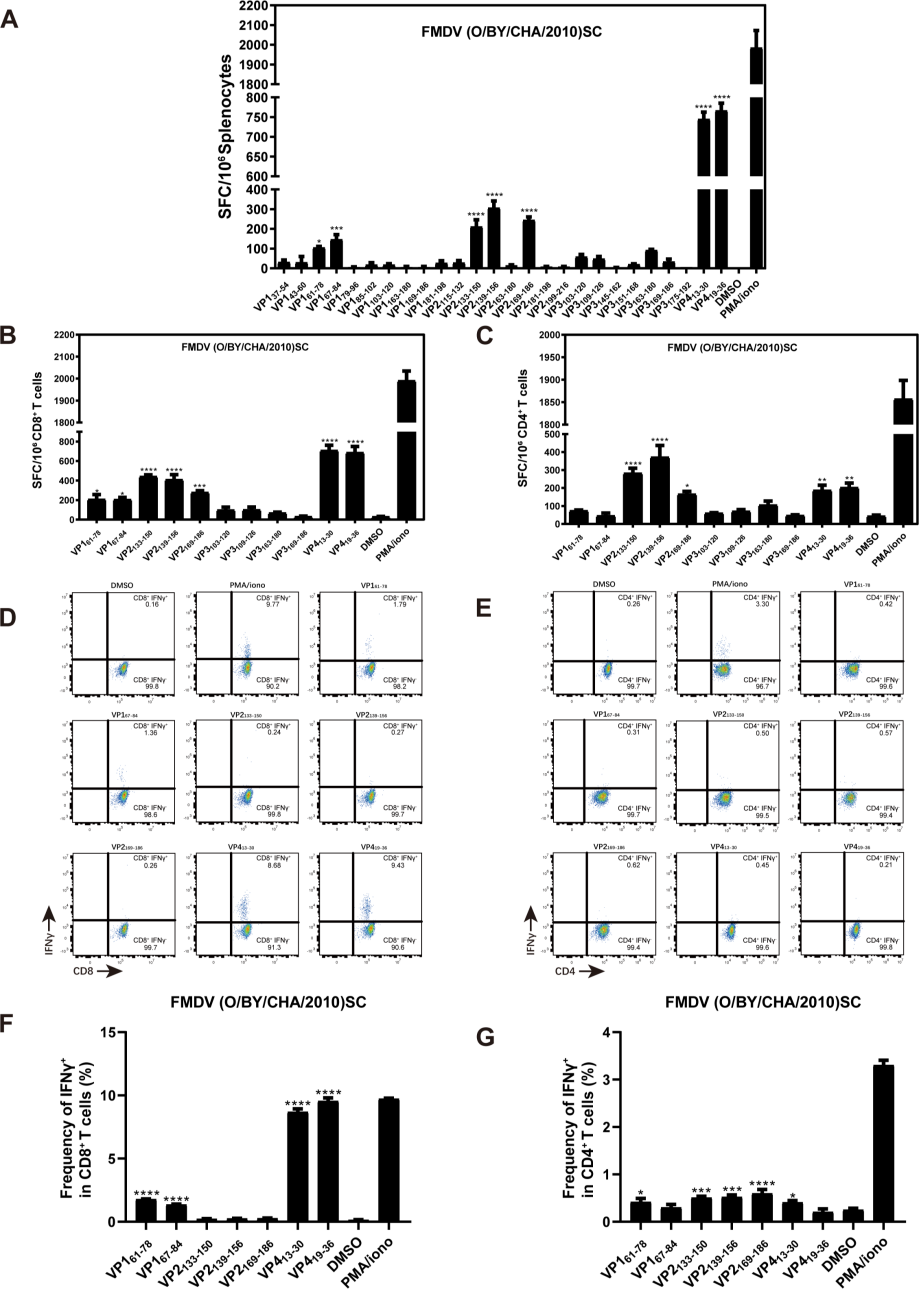
Identification of FMDV epitopes recognized by T cells by IFNγ-ELISPOT and ICS. Five-week-old *Ifnar*^–/–^ mice were infected with 5,000 PFUs of FMDV. (**A**) IFNγ-ELISPOT was conducted using splenocytes isolated from infected mice. A total of 114 peptides from FMDV were screened. (**B, C**) IFNγ-ELISPOT was performed to confirm the presenting type of positive peptides by using (**B**) CD8^+^ or (**C**) CD4^+^ T cells isolated from infected mice. Two independent experiments (*n* = 5 mice for each experiment) were performed in triplicate per peptide. The data are expressed as the mean of spot forming cells (SFC) per 10^6^ splenocytes (**A**), CD8^+^ (**B**), or CD4^+^ T cells (**C**). (**D–G**) ICS was performed to determine IFNγ production at 7 dpi, with positive peptides identified in IFNγ-ELISPOT assay. (**D, F**) showing the frequency of IFNγ^+^ CD8^+^ T cells in scatter diagrams (**D**) and histograms (**F**). (**E, G**) showing the frequency of IFNγ^+^ CD4^+^ T cells in scatterplot diagrams (**E**) and histograms (**G**). Error bars are represented as SEM. One-way ANOVA was used to compare the mean of each peptide with the control (DMSO) Statistical significance is represented by asterisks (*P < 0.05; **P < 0.01; ***P < 0.001; ****P < 0.0001).

To better understand the impact of CMSS1 on antigen presentation, we first needed to establish methods that could accurately reflect the function of antigen presentation and T-cell responses to FMDV. First, to characterize the immunodominant T cell response induced by FMDV, we identified the epitopes from FMDV structural proteins (VP1, VP2, VP3, and VP4) that are recognized by T cells. Next, a total of 114 peptides covering all structural proteins of FMDV were chemically synthesized, and an IFNγ-(Enzyme-Linked ImmunoSpot) ELISpot assay was performed to individually screen the peptides using splenocytes from FMDV-infected mice. Notably, 26 peptides tested positive for FMDV, with seven of these peptides showing significant differences compared to DMSO controls (Fig. 5A). To further characterize the specificity of the MHC-restricted T cell response, we evaluated these seven identified peptides using the IFNγ-ELISPOT assay, focusing on CD8^+^ or CD4^+^ T cells. The results indicate that VP2_133-150_, VP2_139-156_, and VP2_169-186_ induced MHC class I-restricted CD8^+^ T cell responses and MHC class II-restricted CD4^+^ T cell responses with similar capacities. In contrast, VP1_61-78_, VP1_67-84_, VP4_13-30_, and VP4_19-36_ predominantly activated MHC class I–restricted CD8^+^ T cell responses (Fig. 5B and C). Subsequently, intracellular cytokine staining (ICS) was performed to further validate the epitopes identified in the IFNγ-ELISPOT assay. FMDV-primed splenocytes were isolated and stimulated with these seven peptides individually, and the CD8^+^IFNγ^+^ and CD4^+^IFNγ^+^ double-positive cells were quantified (Fig. 5D through G). Consistent with the IFNγ-ELISPOT results, VP1_61-78_, VP1_67-84_, VP4_13-30_, and VP4_19-36_ led to remarkable activations of CD8^+^IFNγ^+^ T cells, especially VP4_13-30_ and VP4_19-36_, with the frequency of IFNγ-producing CD8^+^ T cells reaching approximately 9% (Fig. 5D and F). Moreover, VP2_133-150_, VP2_139-156_, and VP2_169-186_ induced high frequencies of IFNγ-expressing CD4^+^ T cells (Fig. 5E and G). Overall, all seven peptides identified through the IFNγ-ELISPOT assay elicited high frequencies of either CD8^+^IFNγ^+^ or CD4^+^IFNγ^+^ T cells. Finally, we used a bioinformatics program to examine the four FMDV structural proteins (VP1, VP2, VP3, and VP4) (35). Notably, the top-ranked core sequence, which is predicted to possess high binding affinities to MHC molecules, was included in VP1_61-78_ and VP1_67-84_ for H-2Kb, VP2_133-150_, and VP2_139-156_ for H-2IAb, and VP4_13-30_ and VP4_19-36_ for H-2Db. This result further supports our findings described above (Table S1). In conclusion, we identified and screened seven peptides linked to four immunodominant T-cell epitopes found in the structural proteins of FMDV using the IFNγ-ELISPOT and ICS assays.

### An evaluation system for antigen-presenting capacities

**Fig 6:**
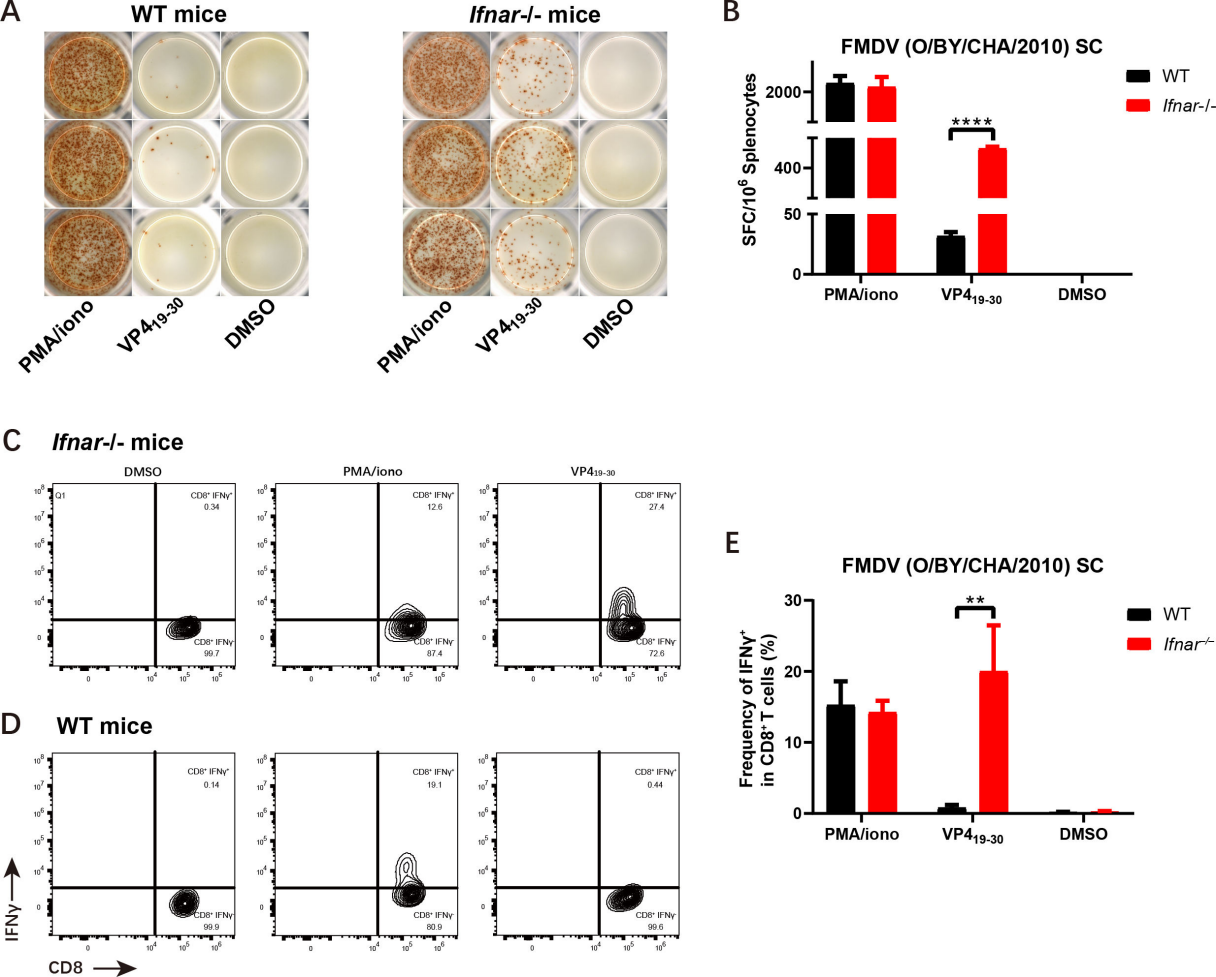
Establishment of an evaluation system for antigen-presenting capacities *in vitro*. Five-week-old WT and Ifnar–/– mice were infected with 5,000 PFUs of FMDV. The peptide VP4_19-30_ was tested using IFNγ-ELISPOT (**A–B**) and ICS (**C–E**) with CD8^+^ T cells isolated from the spleen of infected mice at 7 dpi. Error bars are represented as SEM. A one-way ANOVA was conducted to compare the mean of each peptide with the control (DMSO). Statistical significance is represented by asterisks (**P < 0.01; ****P < 0.0001).

Based on our results from scRNA-seq analysis, the distribution of cDC1 subpopulations was predominantly reduced after FMDV infection. cDC1 cells, being professional antigen-presenting cells, activate CD8 responses. In conjunction with screening for T-cell-specific epitope peptides, we discovered that VP4_13-30_ and VP4_19-36_ can significantly activate T cells, particularly CD8^+^ T cells. Therefore, we opted to synthesize the common region of the two to validate their antigen presentation effects. To this end, we synthesized the overlapped 15 amino acids between these two peptides and subsequently tested the new peptide VP4_19-30_ via IFNγ-ELISPOT and ICS. Not surprisingly, VP4_19-30_ showed intense activity in activating IFNγ-expressing T cells (Fig. 6A through C and E), with 465 ± 10 SFC/10^6^ splenocytes (Fig. 6A and B) and approximately 20% of CD8^+^ T cells expressing IFNγ (Fig. 6C and E). Meanwhile, we also examined the properties of VP4_19-30_ in WT C57 mice, which barely elicited T cell responses (Fig. 6A, B, D and E). The results indicate that VP413-30 is the most effective epitope peptide for activating CD8^+^ T cells. It will also be used in the antigen presentation ability evaluation system during FMDV infection to further assess the regulation of antigen presentation function of Cmss1, as screened in our study.

### Cmss1 antagonizes FMDV-induced MHC class I inhibition and enhances antigen presentation

**Fig 7:**
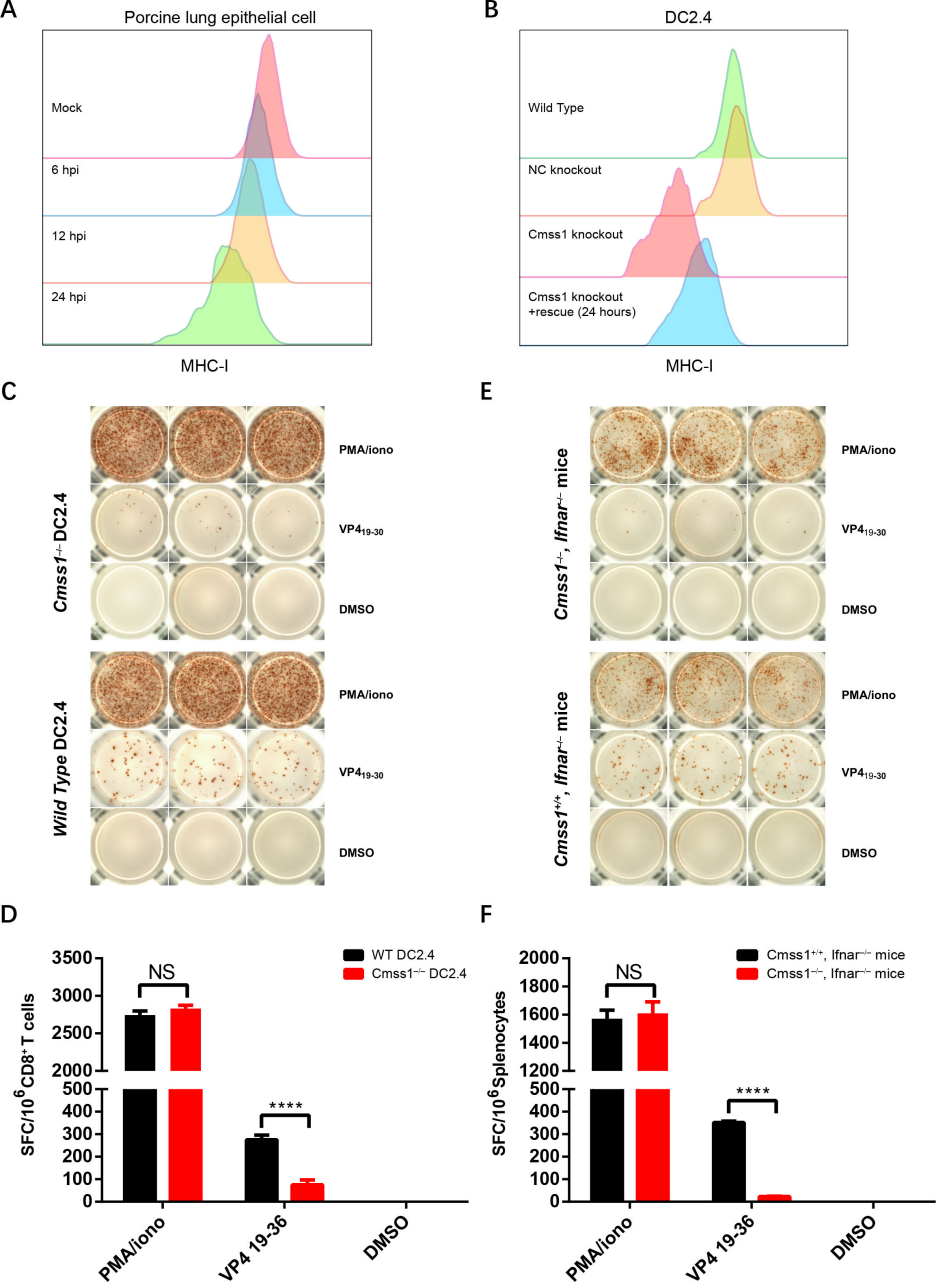
Cmss1 deficiency enhances the inhibitory effects of FMDV on MHC class I-mediated antigen presentation. MHC class I expression was validated during FMDV infection, and the potential regulation of Cmss1 in the antigen processing and presentation process was explored using the evaluation system established in our study. (**A**) Histogram of MHC class I expression levels in porcine lung epithelial cells at 6, 12, and 24 hours post infection. MOI = 1. (**B**) Histogram of MHC class I expression levels in DC2.4 cells under the indicated treatment. (**C–F**) The IFNγ-ELISPOT and evaluation system was used to investigate the function of Cmss1 during antigen processing and presentation. (**C, D**) The Cmss1 knockout DC2.4 cells served as APCs. (**E, F**) Splenocytes isolated from indicated mice were used for the IFNγ-ELISPOT. Statistical significance is represented by asterisks (****P < 0.0001), NS (Not significant or *P* > 0.05).

Since the positive marker VP4_19-30_ described above is associated with the MHC class I-peptide and the inhibition of MHC class I observed during FMDV infection in scRNA-seq (Fig. S5A), we primarily focused on investigating the potential regulation of Cmss1 on MHC class I during FMDV infection. We first tested the MHC class I expression level of porcine lung epithelial cells during FMDV infection by flow cytometry. Notably, MHC class I was found to be downregulated by FMDV in a time-dependent manner (Fig. 7A), which is consistent with previous studies (34). In our scRNA-seq data, Cmss1 exhibited remarkable upregulation during FMDV infection. However, its role in the antigen processing and presentation related to FMDV infection remains unclear. To this end, the Cmss1 knockout DC2.4 cell line was constructed using CRISPR/Cas9 technology, and the knockout efficiency was assessed by Western blot (Fig. S6A and B). A rescue group was established where wild-type DC2.4 was transfected with pcDNA3.1-Cmss1, and flow cytometry was performed to determine the MHC class I expression levels of Cmss1 knockout DC2.4, NC knockout DC2.4 (negative control), wild-type DC2.4, and the Cmss1 rescued DC2.4 group. A dramatic decrease in MHC class I expression levels was observed in the Cmss1 knockout compared to wild-type DC2.4, whereas no such decrease was seen in the NC knockout DC2.4. This effect was partially counteracted in the DC2.4 cells of the Cmss1-rescued group, which were analyzed 24 hours after transfection with the Cmss1 plasmid (Fig. 7B). The results emphasize a crucial role for Cmss1 in the regulatory mechanism of MHC class I.

Based on these results, we further investigated whether Cmss1 could improve the processes of antigen processing and presentation using the evaluation system mentioned above. CD8^+^ T cells from FMDV-infected *Ifnar^–/–^* mice were mixed with Cmss1 knockout and wild-type DC2.4, respectively, stimulated with the positive marker VP4_19-30_, and subsequently subjected to the IFNγ-ELISPOT assay. The Cmss1 knockout DC2.4 group demonstrated significantly fewer positive spots compared to the wild-type DC2.4 group (Fig. 7C and D). The results support the observation that Cmss1 upregulates MHC class I and indicate its ability to enhance antigen processing and presentation during FMDV infection. To further validate this conclusion, *Cmss1^–/–^Ifnar^–/–^* double-knockout mice were constructed using CRISPR/Cas9 technology (Fig. S6C), and the antigen-presenting capacities of VP4_19-30_ were assessed using the evaluation system described above. Consistent with our expectations, compared to *Cmss1^+/+^Ifnar^–/–^* mice, *Cmss1^–/–^Ifnar^–/–^* mice demonstrated reduced capabilities to produce IFNγ during FMDV infection (Fig. 7E and F). Therefore, these results indicate that FMDV inhibits MHC class I-mediated CD8^+^ T cell responses, a process that Cmss1 antagonizes. This suggests that Cmss1 may function as a host molecule in antiviral immunity during FMDV infection, limiting viral infection by enhancing antigen presentation.

### Cmss1 deficiency enhances FMDV pathogenicity in mice.

**Fig 8:**
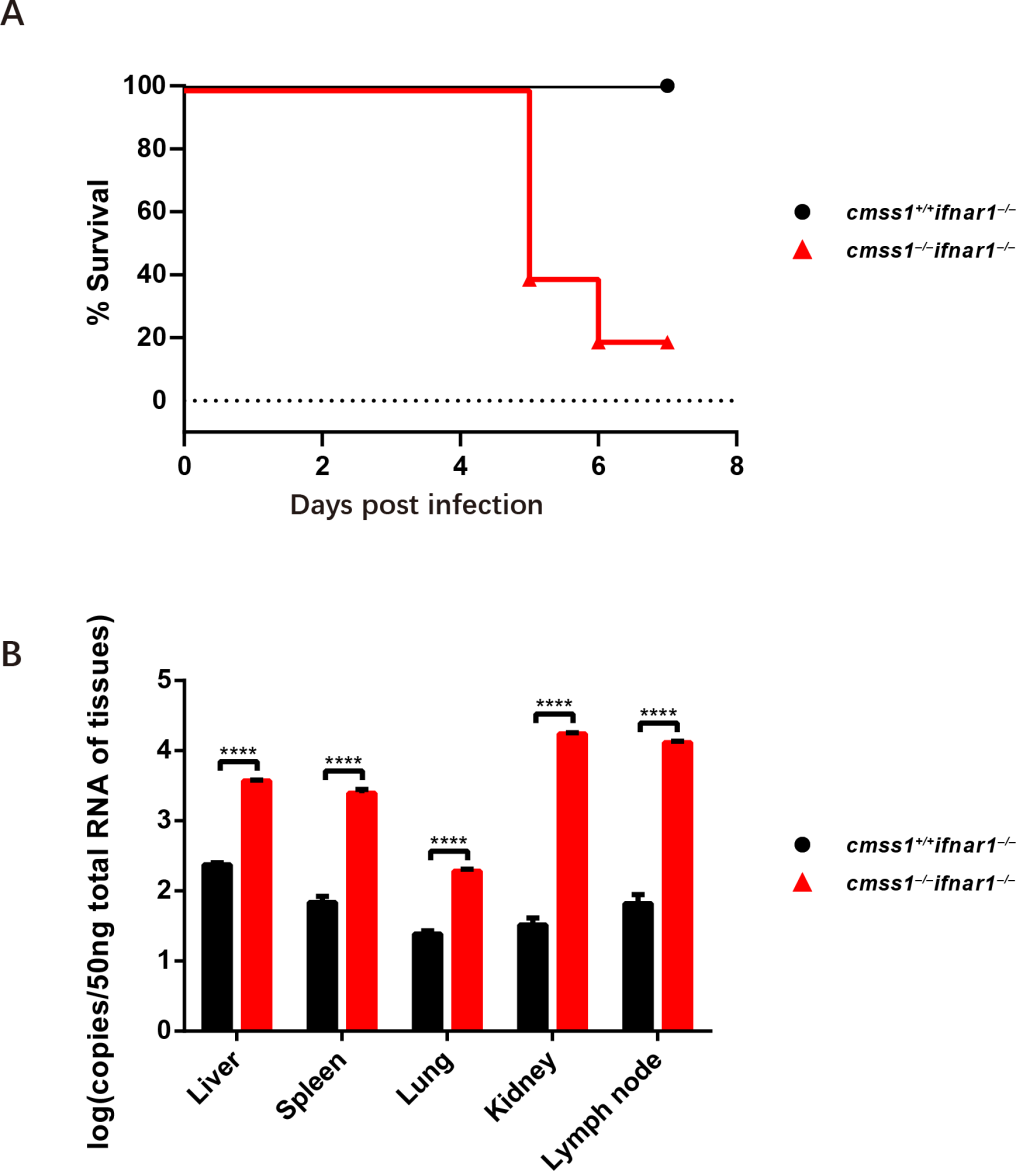
*Cmss1^–/–^* mice are less resistant to FMDV infection. *Cmss1^–/–^Ifnar*^–/–^ and *Cmss1*^+/+^*Ifnar*^–/–^ mice at 4 weeks of age were infected with 10^3^ PFUs of FMDV. Indicated organs were harvested at day 7 post-infection, and the levels of FMDV RNA were determined. (**A**) Survival curves of infected *Cmss1^–/–^Ifnar*^–/–^ (red triangles) and *Cmss1*^+/+^*Ifnar*^–/–^ mice (black circles) (*n* = 5). (**B**) FMDV RNA loads in the heart, peripheral blood, liver, lymph node, spleen, lung, and kidney of *Cmss1^–/–^Ifnar*^–/–^ (red triangles) and *Cmss1*^+/+^*Ifnar*^–/–^ mice (black circles) were quantified using RT-qPCR (*n* = 3). Statistical significance is represented by asterisks (****P < 0.0001).

As Cmss1 has been shown to enhance antigen processing and presentation by mediating MHC class I, we hypothesized that deleting Cmss1 would increase FMDV infection. To test this hypothesis, we evaluated the resistance of *Cmss1*^+/+^*Ifnar*^–/–^ and *Cmss1*^–/–^*Ifnar*^–/–^ mice to FMDV infection by injecting 10^3^ PFUs of FMDV subcutaneously into each mouse. The results indicated that *Cmss1^–/–^Ifnar^–/–^* mice were significantly less resistant to FMDV infection than *Cmss1^+/+^Ifnar^–/–^* mice. Infection with 10^3^ PFUs of FMDV caused death at 5 dpi in *Cmss1^–/–^Ifnar^–/–^* mice, whereas it did not result in death in *Cmss1^+/+^Ifnar^–/–^* mice (Fig. 8A). Additionally, we analyzed the viral RNA from the organs of *Cmss1^+/+^Ifnar^–/–^* and *Cmss1^–/–^Ifnar^–/–^* mice, discovering that knocking out Cmss1 led to an increase in viral RNA levels by approximately 20, 50, 7.5, 600, and 190 times in the liver, spleen, lung, kidney, and lymph node, respectively (Fig. 8B). These results further demonstrate that Cmss1 antagonizes FMDV infection and reduces viral pathogenicity by enhancing host antigen presentation function.

## DISCUSSION

FMD is a severe, highly contagious viral disease affecting livestock, which has a considerable economic impact. However, the mechanisms by which FMDV prolongs infection *in vivo,* and the responses of host cells to infection remain largely unclear. Since DCs coordinate both the innate and adaptive immune systems, gaining a deeper understanding of DC-related biological processes during FMD enhances our knowledge of the underlying interactions between the host and FMDV. In this study, scRNA-seq was conducted to comprehensively characterize the cell distributions and gene expression profiles of DCs derived from the immune cells in the spleens of FMDV-infected subjects. In search of potentially novel host factors involved in antigen processing and presentation, we established an evaluation system by identifying immunodominant T cell epitopes derived from FMDV structural proteins. Finally, we conclude that FMDV infection alters the proportions of DC subsets and impairs their antigen processing and presentation functions. Importantly, we identified cmss1 as a novel host factor; its depletion in DC2.4 cells and mice resulted in a significant decrease in MHC class I, which inhibited the CD8^+^ T cell response and ultimately reduced resistance to FMDV infection.

The interactions between viruses and DCs remain a vibrant area of research. Many viruses engage with the biological processes of DCs, thereby enhancing their replication. The Ebola virus VP35 and the gamma 1 34.5 protein of herpes simplex virus 1 impair dendritic cell maturation (36, 37). The Epstein-Barr virus inhibits DC development by promoting the apoptosis of their monocyte precursors in the presence of granulocyte-macrophage colony-stimulating factor and interleukin-4 (38). The measles virus suppresses cell-mediated immunity by interfering with the survival and functions of dendritic cells and T cells (39). In our scRNA-seq data, FMDV suppresses the MHC class I and II molecules of DCs during infection. These phenotypes have previously been observed in murine DCs (40) and PK-15 and ESK-4 cell lines (34). Our study also reveals the reprogramming of DC subsets during FMDV infection, which aligns with that of varicella zoster virus during its natural infection (41). Type 1 immune responses target intracellular pathogens, such as viruses, requiring IFN-γ-activated cytotoxic CD8^+^ T cells for clearance. Numerous studies have shown that cDC1s are crucial for type 1 responses due to their ability to activate CD8^+^ T cells (42–46). Based on our data, the decrease in the proportion of cDC1 indicates that FMDV may enhance its productive infection by impairing cDC1s, which are crucial for Type 1 immune responses.

Our data from murine spleens also indicate significant downregulation of ribosome-related functions. Interestingly, a recent study has demonstrated high-efficiency induced ribosomal frameshifting by the West Nile Virus (47). Viruses regulate gene expression by manipulating programmed ribosomal frameshifting (PRF), which alters the mRNA reading frame of ribosomes during translation. Furthermore, a recent study on murine norovirus also suggests a downregulation of ribosome biogenesis in infected cells at single-cell resolution (48). These studies suggest that manipulating ribosomes may be crucial for virus and host infection. However, despite the overall reduction in ribosome-related processes, Cmss1 displays a unique trend during FMDV infection. Cmss1 is an RNA-binding protein that acts as a component of the small ribosomal subunit, contributing to translation (49). Its expression has been observed in various tissues, including those involved in immune responses. Gene enrichment analyses indicate that Cmss1 primarily participates in ribonucleoprotein complex biogenesis, rRNA metabolism, ncRNA processing, translation initiation, RNA localization, and RNA catabolism (50). While Cmss1 primarily functions in ribosomal activity and protein synthesis, its expression patterns and associations with immune-related tissues suggest it may play a potential role in immune responses. For instance, a recent study indicates that Cmss1 is a novel host factor in maintaining HIV-1 latency (51). However, the precise role of Cmss1 in influencing immunity has yet to be fully clarified. Considering the clinically latent infection of FMDV, Cmss1 may differ from other ribosome-related genes and play a vital role in antigen processing and presentation.

To explore the potential function of Cmss1 in antigen processing and presentation, we provide a validated map of the T cell response to FMDV structural proteins, identifying four epitopes. Among these, the most effective epitope is the CD8^+^ T cell epitope, indicating the critical role of the CD8^+^ T cell response in FMDV infection. These findings are supported by those of other research. For instance, studies have demonstrated that enhancing antigen-specific CD8^+^ T cell responses can contribute to early protection against FMDV (52). Additionally, specific viral peptides displayed by MHC class I molecules stimulate the activation and proliferation of CD8^+^ T cells, resulting in the targeted destruction of infected cells (53). Since VP4_13-30_ and VP4_19-36_ activated IFNγ-secreting T cells most effectively, we established a system to evaluate the antigen-presenting capabilities of DCs by using overlapping 15 amino acids (VP4_19-30_) as a positive marker for activating CD8^+^ T cells. Using this tool, we validate the auxo-action of Cmss1 in the MHC class I-dependent antigen processing and presentation processes. CD8^+^ T cell responses play a crucial role in antiviral immunity, acting rapidly before the humoral immune response is completely established. These cells secrete cytokines like IFN-γ, which enhance antiviral immune responses. They also exert cytotoxic effects by releasing perforin and granzymes, inducing apoptosis, and limiting viral replication. The CD8^+^ T cell response often precedes the antibody immune response, enabling a timely reaction to viral infections and helping resist them before neutralizing antibodies are produced. Additionally, the CD8^+^ T cell-mediated immune response is crucial for the latency and persistence of infections as the body requires a continuous cellular immune response for adequate protection. Moreover, CD8^+^ T cells contribute to long-term immunity by differentiating into memory T cells, which facilitate a quicker and more vigorous response upon subsequent exposure to the same virus, thereby lessening disease severity. This memory function plays a crucial role in vaccine-induced protection. Consequently, CD8^+^ T cell responses are essential for both immediate viral clearance and long-term immune protection, forming a fundamental part of the host’s defense against viral pathogens. Currently, FMDV-inactivated vaccines exhibit good efficacy but do not induce early immune responses in vaccinated animals. In our study, Cmss1 has been shown to regulate MHC-I-dependent CD8^+^ T cell antigen presentation during FMDV infection. This indicates that Cmss1 is crucial in guiding early immune activation and promoting long-term immunity in FMDV vaccine development. To further explore the underlying mechanism of the auxo-action of Cmss1 in MHC class I-dependent antigen processing and presentation, we constructed a *Cmss1^–/–^* DC2.4 cell line and *Cmss1^–/–^* mice. Using these, we proved that Cmss1 significantly upregulates MHC class I in DCs. Importantly, *Cmss1*^–/–^ mice are less resistant to FMDV infection than their *Cmss1*^+/+^ littermates, exhibiting notable clinical symptoms and elevated virus titers in multiple organs. These findings present a valuable new gene not previously identified as involved in FMDV infection and the antigen presentation process.

In summary, our findings confirm the reprogramming of DC subsets and suppression of antigen processing and presentation in DCs during FMDV infection, as revealed by scRNA-seq. This method is a powerful means of assessing the antigen-presenting functions of cells concerning FMDV infection and highlights Cmss1 as a promising target for future defenses against FMD. It is important to note that identifying functionally relevant molecular targets involved in antigen processing and presentation is a significant challenge in the field. Clearly, our understanding of the molecular mechanisms involved in the interplay between FMDV and its host is still inadequate. Therefore, further investigation of various infection samples from different periods, including immune tissue samples from both acute and chronic stages as well as from recovering animals, will comprehensively assess the immune characteristics of FMDV infection and identify valuable host molecules that may be crucial for developing a more robust strategy for the prevention and control of FMDV infection.

## MATERIALS AND METHODS

### Mice, virus, and cells

WT C57BL/6 mice were bred at the Lanzhou Veterinary Research Institute, Chinese Academy of Agricultural Sciences (LVRI, CAAS). Ifnar KO mice (*Ifnar*^–/–^) and Cmss1 KO mice (*Cmss1^–/–^, Ifnar^–/–^*) were obtained from Cyagen Biosciences Inc. Animals were bred from heterozygous parents and validated using PCR and Western blot. The FMDV/O/GD/CHA/2010 /S/BF8 strain virus was stored at LVRI, CAAS. DC2.4, HEK293T cells, and porcine lung epithelial cells were stored in the laboratory.

### Generation of knockout DC2.4 by CRISPR/Cas9 technology

HEK293T cells were initially transfected with lentivirus plasmid and packaging assistant plasmids psPAX2 and pMD2.G for 48 h. After that, the viral supernatant was collected, and the virus was concentrated through ultracentrifugation. Subsequently, the wild-type DC2.4 cells (2 × 10^6^ cells/well in 6-well plates) were transfected using lentivirus specific to the indicated target genes. After 24 hours of infection, the positive cells were screened by adding 5 µg/mL of puromycin before the cloning selection. All selected clones were amplified and sequenced for further use. Genomic DNA was extracted from the edited cells using the MiniBEST Universal Genomic DNA Extraction Kit (TaKaRa, 9765, Dalian, China) following the manufacturer’s protocol, and 50 ng of genomic DNA from each clone was utilized to amplify the region surrounding the sgRNA cleavage site. The PCR was performed using a Bio-Rad C1000 thermal cycler. The final products were visualized on a 1% agarose gel and then sequenced. This research used the published mouse genome sequence and gene annotation information (*Mus musculus*) to design sgRNAs based on the reference standards (54).

### Animal infection

All animals were handled in strict accordance with good animal practice according to the Animal Ethics Procedures and Guidelines of the People’s Republic of China, and the study received approval from the Animal Ethics Committee of the Lanzhou Veterinary Research Institute, Chinese Academy of Agricultural Sciences (Approved No. LVRIAEC-2022-082). All animal experiments involving FMDV were conducted in the Biosafety Level 3 (BSL-3) facilities at LVRI, CAAS, and were approved by the Ministry of Agriculture and Rural Affairs and the China National Accreditation Service for Conformity Assessment. Female mice aged 4 to 6 weeks were used in this study, and all *in vivo* infections were performed through subcutaneous inoculations with 100 µL of 10% FBS/RPIM 1640 medium containing the specified PFUs of FMDV. In all experiments, mice that received 10% FBS/RPIM 1640 medium buffer are designated as mock.

### Single-cell RNA-seq

Initially, the spleens were washed with cold RPIM 1640 medium (containing 0.04% BSA) and minced with scissors. Afterward, the tissues were incubated at 37℃ for 1 h with enzymatic hydrolysate containing 3 mg/mL Collagenase I (Gibco, USA). The cell suspensions were filtered twice using a 40 µm filter and then harvested by centrifugation at 300 *g* for 5 minutes. The filtered cells were subjected to red blood cell lysis (MACS, Germany) at 4°C for 10 minutes before collection. Finally, the cells were resuspended in cold RPMI 1640 medium, and the LUNA-FL Counter (Logos Biosystems) was utilized to assess cell viability and cell quantification. The single-cell libraries were generated using the Chromium Next GEM Single Cell 3ʹ GEM, Library & Gel Bead Kit v3.1 (10 × Genomics) and Chip Kit following the manufacturer’s protocol (OE Biotech Co., Ltd. Shanghai, China) (55). The libraries were then sequenced on Illumina Nova 6000 PE150.

### Single-cell RNA-seq data analysis

After undergoing the QC criteria, single splenocytes were subjected to downstream analysis. The Normalize Data function in Seurat (56) and the global-scaling normalization method “Log Normalize” were applied to adjust the library size and the gene expression measurements for each cell by the total expression, multiplied by a scaling factor-10,000 (the results were log-transformed). Moreover, top variable genes across single cells were identified using the previously described method (57), and the FindVariableGenes function (mean.function = Fast Exp Mean, dispersion.function = Fast Log VMR) in Seurat (56) was used for selection. To reduce dimensionality using the RunPCA function in Seurat (56) and cluster cells according to their gene expression profiles, principal component analysis (PCA) and graph-based clustering (by using the FindClusters function in Seurat (56)) were performed. Then, the 2-dimensional Uniform Manifold Approximation and Projection (UMAP) algorithm with the Run UMAP function in Seurat (56) was used to visualize cells, and marker genes of each cluster were identified via the FindAllMarkers function (test. use = presto) in Seurat (56). FindAllMarkers identified positive markers for a given cluster in comparison to all other cells. We then utilized the R package Single R (58) (version 1.4.1), a novel computational method for unbiased recognition of cell types in scRNA-seq, along with the reference transcriptomic data set “scmca” (59) to infer the originating cell of each single cell independently and identify their cell types. Differentially expressed genes (DEGs) were identified using the FindMarkers function (test. use = presto) in Seurat (56). A P value of < 0.05 and |log2foldchange| > 0.58 were established as the thresholds for significant differential expression. Gene Ontology (GO) enrichment and KEGG pathway enrichment analyses of DEGs were performed using R according to the hypergeometric distribution.

### Flow cytometry (FACS)

For flow cytometry analysis, cells were counted using the Countess 3 FL (Invitrogen, USA) and washed twice with 500 µL of flow cytometry staining buffer (Biosharp, BL1136A). Murine cells were labeled with anti-CD16/CD32 (BioLegend, 101302) and treated with the specified antibodies, while swine cells were treated with the specified antibodies immediately. The Zombie Aqua Fixable Viability Kit (BioLegend, 423102) was subsequently used according to the manufacturer’s protocol. Finally, the stained cells were washed twice, resuspended in 200 µL of flow cytometry staining buffer, and collected for analysis using the CytoFlex LX (Beckman, Germany) and FlowJo software (BD Biosciences). The antibodies used for flow cytometry analysis in this study are listed as follows. FITC-conjugated SLA class I (Bio-Rad, MCA2261), PE-conjugated MHC class I (Invitrogen, 12-5958-82), PE594-conjugated CD45 (BioLegend, 103146), AF700-conjugated CD3 (BioLegend, 100216), Pacific Blue-conjugated CD4 (BioLegend, 100531), PE-conjugated CD8a (BioLegend, 100708), PEcy7-conjugated CD19 (BioLegend, 115519), PerCP-conjugated I-A/I-E (BioLegend, 107626), AF488-conjugated CD11c (BioLegend, 117311), AF647-conjugated F4/80 (BioLegend, 123122), and BV605-conjugated CD11b (BioLegend, 101257).

### Immunohistochemistry (IHC)

The heart and spleen tissues were collected at the specified time and fixed in 4% paraformaldehyde. Following this, all tissues were dehydrated, paraffin-embedded, sectioned at 4 µm, and stained with hematoxylin and eosin (H&E).

### RNA extraction and reverse transcription-quantitative polymerase chain reaction (RT-qPCR)

Total RNA was extracted from heart, peripheral blood, liver, lymph nodes, spleen, lung, and kidney tissues using TRIzol reagent (TaKaRa, 9766, Dalian, China). It was then reverse-transcribed into complementary DNA (cDNA) with a PrimeScript RT reagent kit (TaKaRa, RR036A, Dalian, China) according to the manufacturer’s instructions. The cDNA underwent quantitative analysis using the Light Cycler 480 II System (Roche, Basel, Switzerland), utilizing Probe qPCR Mix with UNG (TaKaRa, RR391A, Dalian, China). The primers used in this study are listed in Table 2.

**TABLE 2:** Oligonucleotides used for cloning sgRNA and detecting qPCR

Primer	Name	Sequence (5’−3’)
3D (Taqman qPCR)	3D-F	ACTGGGTTTTACAAACCTGTGA
	3D-R	GCGAGTCCTGCCACGGA
	TaqMan probe	FAM-TCCTTTGCACGCCGTGGGAC-TAMRA
Mouse-Cmss1-sgRNA KO cell	Cmss1-sgRNA	GAGACTGTGAGTCAAATCAT-TGG
Mouse-Cmss1-gRNA KO mice	Cmss1-gRNA-A1	TCCTTCAAAGCCGATCCTTC-AGG
	Cmss1-gRNA-A2	GGTTTGCCTCCTGCCGAGCA-GGG
	Cmss1-gRNA-B1	TGATGCAACATCTAGAAATT-GGG
	Cmss1-gRNA-B2	GAGCGTCTGCCTTTAACTTA-TGG

### Peptide synthesis

All peptides were synthesized by Nanjing TG peptide Biotechnology Co., Ltd on the 10 mg scale, and mass spectral analysis was performed for each peptide to validate the synthesis. All peptides were purified by reverse-phase HPLC to ≥ 95% purity and dissolved in DMSO.

### Western blot analysis

The cell precipitate was lysed in RIPA Lysis Buffer (Beyotime, P0013C) supplemented with 1% protease inhibitor cocktail (NCM Biotech, P001). A BCA Protein Assay kit (Thermo Fisher Scientific, A55864) was used to determine the total protein concentration. The samples were analyzed by SDS-PAGE and transferred to polyvinylidene difluoride (PVDF) membranes. The membranes were blocked with 5% non-fat milk and then incubated with primary antibodies, followed by incubation with HRP-conjugated secondary antibodies. Immunoreactive proteins were detected using an imaging analysis system (Amersham Imager 600; GE, USA). The antibodies used for Western blot analysis in this study are listed as follows. Cmss1 primary antibody (Abmart, PU758791S) and tubulin primary antibody (Proteintech, 66240-1-Ig).

### ICS

For ICS, splenocytes were counted using Countess 3 FL (Invitrogen, USA) and washed with 500 µL of flow cytometry staining buffer (Biosharp, BL1136A) twice. The splenocytes were then resuspended in 10% FBS/RPMI 1640 medium. After that, the splenocytes were seeded in 96-well plates at a density of 10^6^ cells per well and cultured in 10% FBS/RPMI 1640 medium with the indicated individual peptide (10 µg/mL) for 1 hour. Positive (PMA-ionomycin) and negative (DMSO) controls were included in all experiments. Splenocytes were treated with 1 × brefeldin A (Biolegend, 420601, USA) for 5 hours and then labeled with anti-CD16/CD32 (BioLegend, 101302), PE594-conjugated CD45 (BioLegend, 103146), AF700-conjugated CD3 (BioLegend, 100216), Pacific Blue-conjugated CD4 (BioLegend, 100531), PE-conjugated CD8a (BioLegend, 100708), and Zombie AquaTM Fixable Viability Kit (BioLegend, 423102). Cells were subsequently fixed and permeabilized using the Fixation and Permeabilization kit (Invitrogen, GAS0003, USA) following the manufacturer’s protocol, and PE-Cy7-conjugated anti-IFN gamma (MBL, 60-7311-U100) was employed to label IFNγ^+^ cells. Cells were finally collected and analyzed using CytoFlex LX (Beckman, Germany) and FlowJo software (BD Biosciences).

### *In vitro* IFNγ ELISPOT

CD8^+^ and CD4^+^ T cells were isolated using magnetic bead positive selection (Miltenyi Biotec, Germany). A total of 2 × 10^5^ splenocytes, specifically CD8^+^ or CD4^+^ T cells, were stimulated with the indicated individual peptide (10 µg/mL) along with 10^5^ DC2.4 cells, which served as antigen-presenting cells (APCs) in the 96-well Mouse IFN-γ precoated ELISPOT kit (Dakewe, 2210003, China) in triplicate. The IFNγ-ELISPOT assay was conducted as per the manufacturer’s protocol. Positive (PMA-ionomycin) and negative (DMSO) controls were included in all experiments.

### Cell viability assay

The potential cytotoxicity of compounds against DC2.4 was assessed using the Cell Counting Kit-8 (CCK-8) (Biosharp, BS350B, China), following the manufacturer’s instructions. Initially, DC2.4 was seeded into 96-well plates at a density of 5 × 10^3^ cells per well and cultured in 5% FBS/RPIM 1640 medium for 12 h. Then, the specified compounds were serially diluted by a factor of 10 in 5% FBS/RPIM 1640 medium and added to the cells. Control wells contained 5% FBS/RPMI 1640 medium with 0.1% DMSO. After incubating for 24 hours at 37°C in a 5% CO2 incubator, 10 µL of CCK-8 reagent was added to each well and incubated for an additional 4 hours at 37°C, after which the absorbance at 450 nm was measured using an ELISA reader.

### RNA velocity analysis

To conduct the RNA velocity analysis, the spliced reads and unspliced reads were recounted using the Python script velocyto.py (33) (https://github.com/velocyto-team/velocyto.py) on the Cell Ranger output folder. The calculation of RNA velocity values for each gene in each cell and the embedding of RNA velocity vectors into a low-dimensional space were performed using the R package velocyto.R[1] v0.6. Velocity fields were projected onto the t-SNE embedding obtained from Seurat.

### Pseudotime analysis

The developmental pseudotime was determined using the Monocle2 package (60). The raw count was initially converted from a Seurat object to a CellDataSet object using the importCDS function in Monocle. The differentialGeneTest function from the Monocle2 package was employed to select ordering genes (qval < 0.01) that were likely informative for ordering the cells along the pseudotime trajectory. Dimensional reduction clustering analysis was conducted with the reduceDimension function, followed by trajectory inference using the orderCells function with default parameters. Gene expression was visualized using the plot_genes_in_pseudotime function to monitor changes over pseudo-time.

### Statistical analysis

All data were analyzed using GraphPad Prism software (version 8.0). Results were presented as the mean ± standard errors, and a *P* value < 0.05 was considered significant.

## Data Availability

The data that support the findings of this study are openly available in the GEO repository at https://www.ncbi.nlm.nih.gov/geo/ and can be found under accession number GSE279768.

## SUPPLEMENTAL MATERIAL

Supplemental material jvi.01249-25-s0001.pdf Figures S1 to S6; Table S1.

ASM does not own the copyrights to Supplemental Material that may be linked to, or accessed through, an article. The authors have granted ASM a non-exclusive, world-wide license to publish the Supplemental Material files. Please contact the corresponding author directly for reuse.
