# Supplementary material for: Cmss1 limits FMDV infection by enhancing antigen presentation and CD8+ T cell responses
Source: J Virol. 2025 Nov 24;99(12):e01249-25. doi: 10.1128/jvi.01249-25 (PMC12724260; doi:10.1128/jvi.01249-25)
Supplement: Supplemental material — Figures S1 to S6; Table S1. [file jvi.01249-25-s0001.pdf]

Supplemental material

Cmss1 limits FMDV infection by enhancing antigen presentation and CD8$^+$ T cell responses

Yang Wang[1,2], Lihong Zhang[1,2], Jieru Deng[3], Linlin Zheng[1,2], Zhihua Chen[1,2], Zhao Zhang[1,2], Han Zhang[1,2], Jingjing Pei[1,2], Haixue Zheng[1,2]

1 State Key Laboratory for Animal Disease Control and Prevention, College of Veterinary Medicine, Lanzhou University, Lanzhou Veterinary Research Institute, Chinese Academy of Agricultural Sciences, Lanzhou, China

2 Gansu Province Research Center for Basic Disciplines of Pathogen Biology, Lanzhou, China

3 Department of Microbiology and Immunology, The University of Melbourne, at The Peter Doherty Institute for Infection and Immunity, Melbourne, Victoria, Australia

Address correspondence to Jingjing Pei, jpei@caas.cn, or Haixue Zheng, zhenghaixue@caas.cn

Yang Wang, Lihong Zhang, and Jieru Deng contributed equally to this article. Author order was determined by the duration worked on this project.

This PDF file includes:

Figures S1 to S6

Table S1

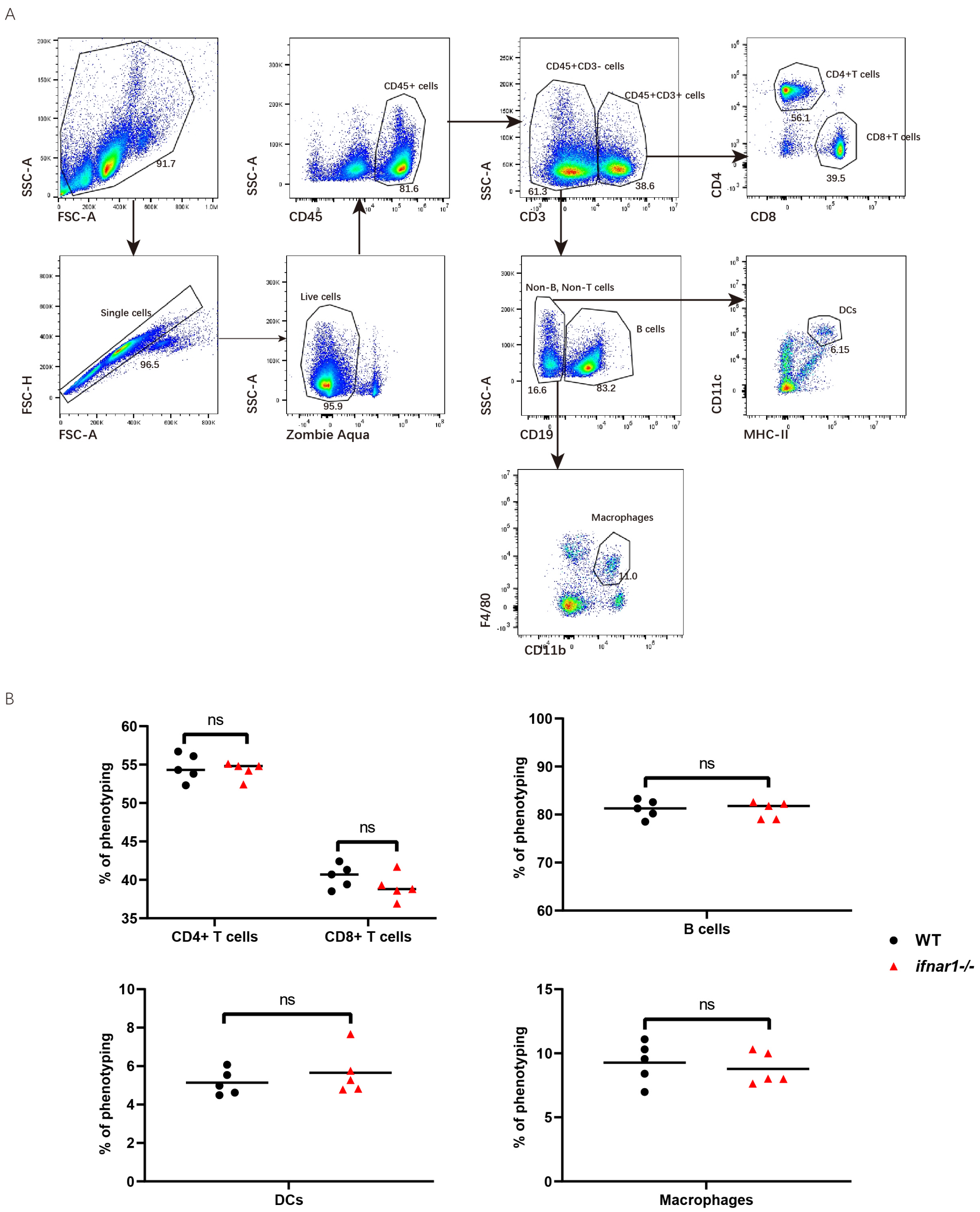

Figure S1. The validation of immune cell distribution in WT and *Ifnar*$^{-/-}$ mouse spleens using FACS

WT and *Ifnar*$^{-/-}$ mice at five weeks of age were infected with 5000 PFUs of FMDV. Splenocytes were isolated at 7dpi and subjected to flow cytometry analysis. (A) The gating strategy for immune cell clustering. (B) The proportions of T cells, B cells, DCs and macrophages in WT and *Ifnar*$^{-/-}$ mouse spleen.

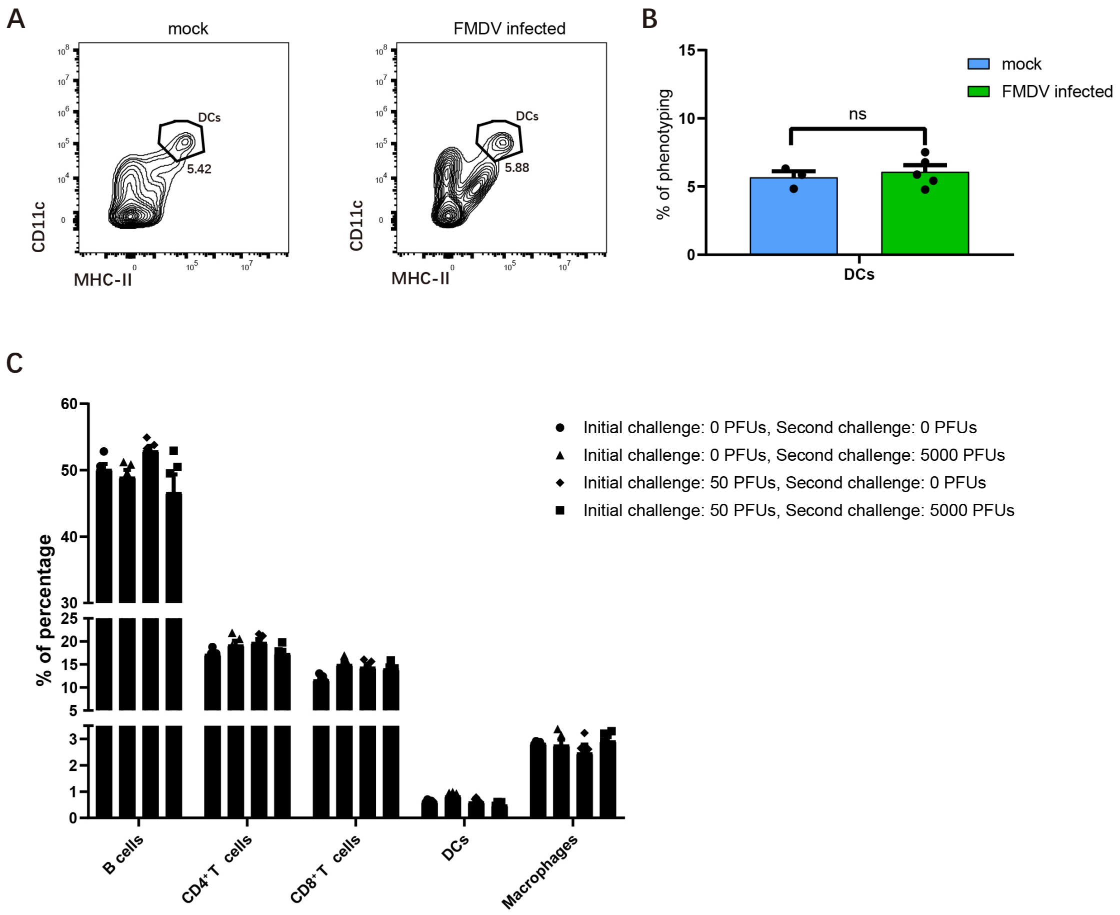

Figure S2. Proportions of DCs in mice and the characteristics of secondary responses to FMDV

(A-B) Five-week-old *Ifnar*$^{-/-}$ mice were infected with 5000 PFUs of FMDV. The proportions of DCs were validated using FACS at 7 dpi and shown in scatter diagrams (A) and histograms (B). (C) Four groups of mice (n=5) were primary and secondary infected with indicated dose of FMDV at day 0 and day 30. The proportions of T cells, B cells, DCs and macrophages in these four groups at 35 dpi were validated using FACS and shown in bar plots.

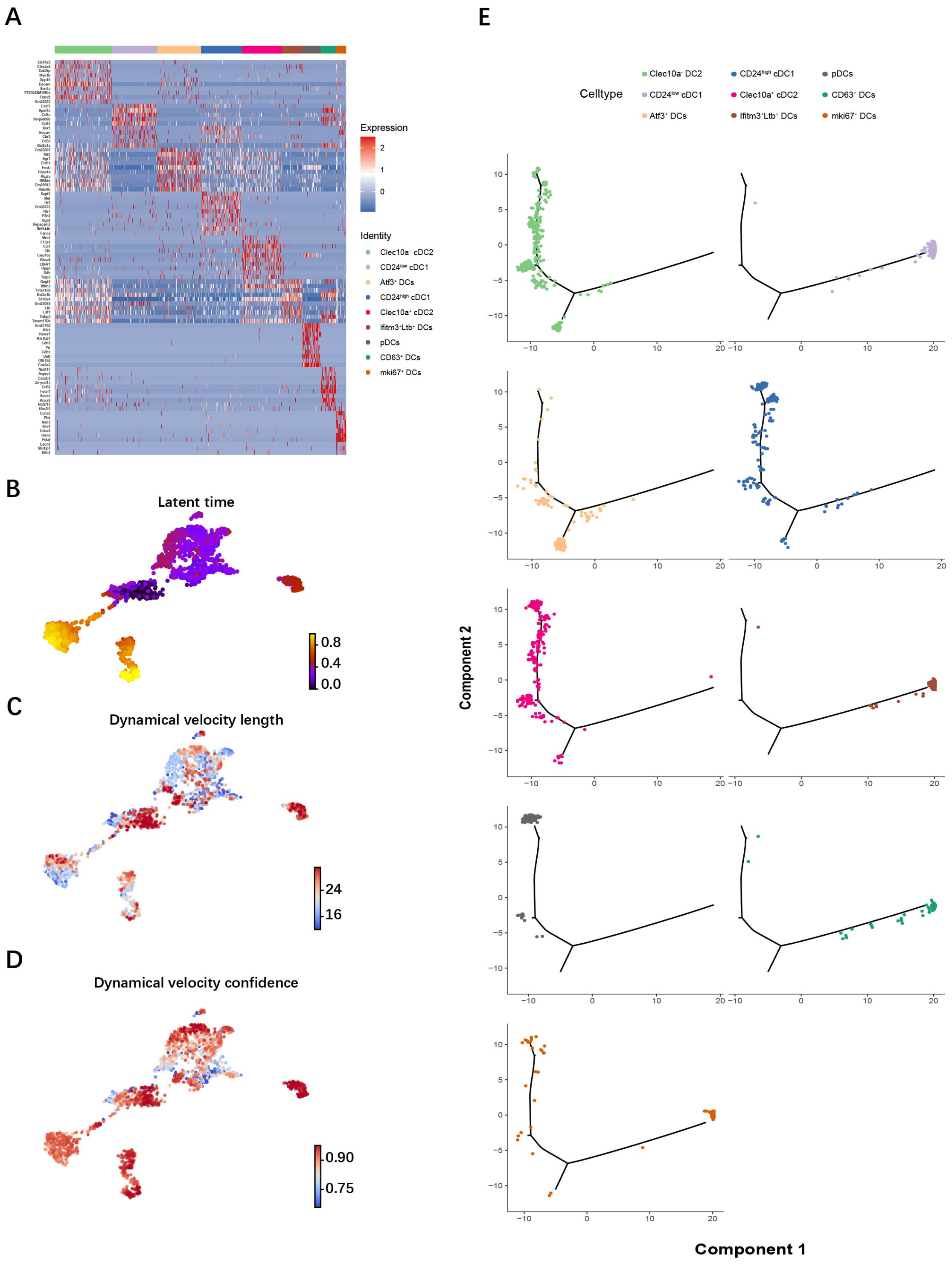

Figure S3. Mapping dendritic cell subsets in mouse spleens during FMDV infection

(A) Markers to which DC subsets clusters are annotated. (B-D) RNA velocity analysis of spleen scRNA-seq data. (B) showing the latent time of cell differentiation, (C) showing the speed of cell differentiation and (D) showing the confidence coefficient.

A

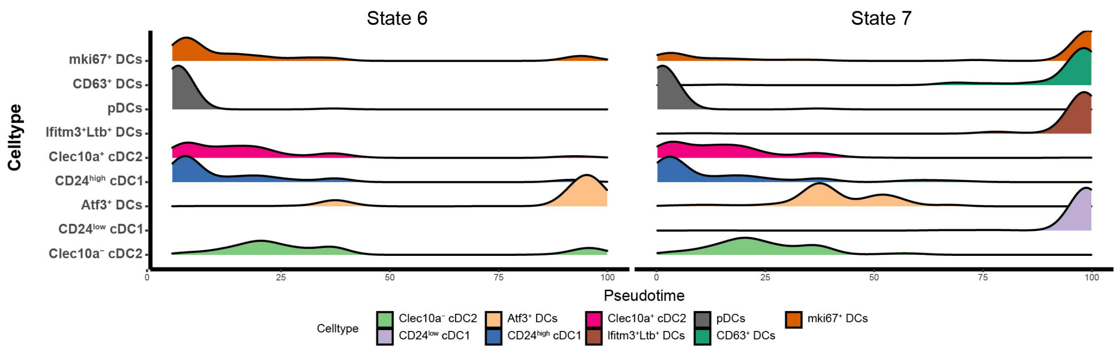

B

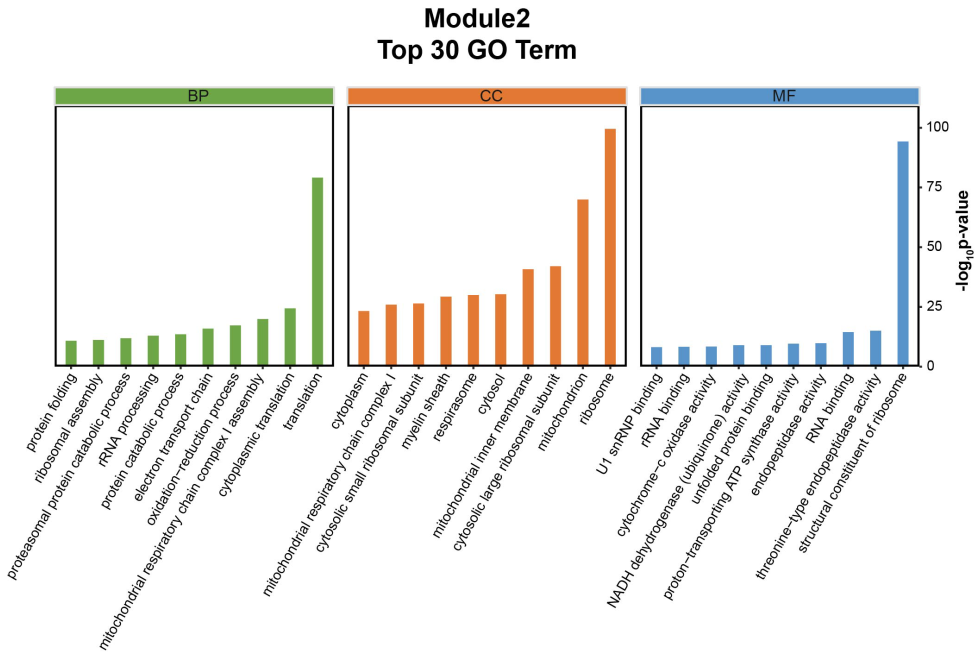

Figure S4. Characteristics of DC proportions and gene expression over pseudotime trajectory.

(A) Ridge Plot showing the cell density of different cell states along the latent time.

(B) GO analysis of different gene expression in module 2 of DCs based on the pseudotime results.

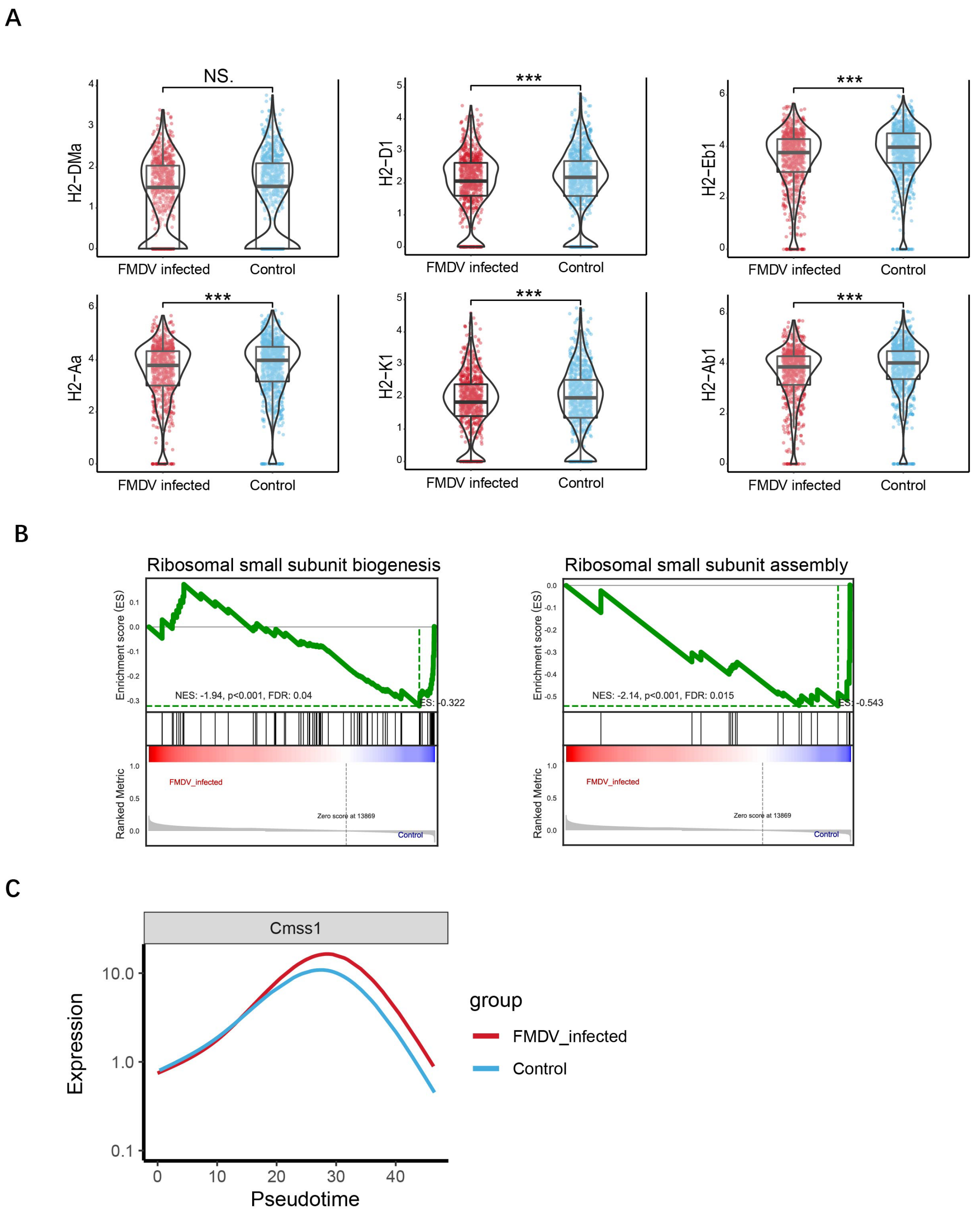

Figure S5. Characterization of DC responses in mice spleens infected with FMDV

FMDV infection induces decreases in MHC and ribosome-related genes and activates Cmss1 expression in DCs. (A) Violin plots showing MHC class I/II expression in DCs from the spleens of FMDV- and mock-infected mice. (B) Changes in ribosome-related pathway activities scored in DCs using GSEA, accompanied by enriched GO terms. (C) Cmss1 expression in DCs from the spleens of FMDV- and mock-infected mice along the pseudotime.

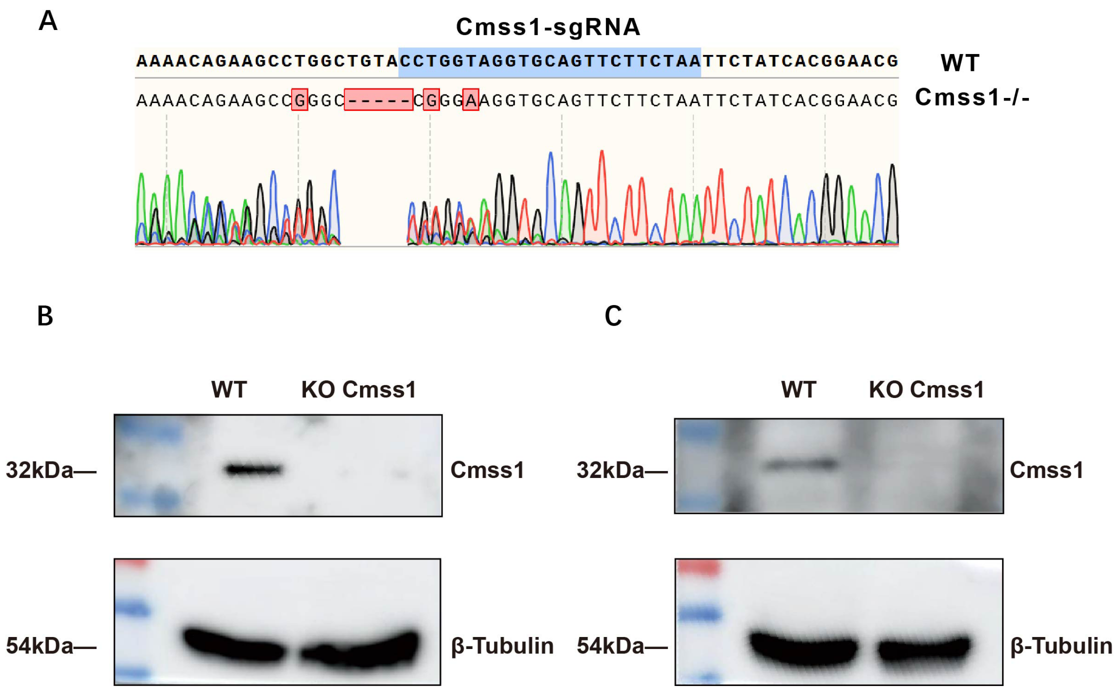

Figure S6. Validation of the Cmss1 knockout DC2.4 cell line and mice

The Cmss1 knockout DC2.4 cell line and mice were developed using CRISPR/Cas9 technology and confirmed by Sanger sequencing and Western blot analysis. (A) Sanger sequencing and (B) Western blot analysis of Cmss1 in mock and Cmss1-knockout DC2.4 cell lines, with Tubulin serving as a loading control. (C) Western blot analysis of cmss1 in $\mathit{Cmss1}^{-/-}\mathit{Ifnar}^{-/-}$ and $\mathit{Cmss1}^{+/+}\mathit{Ifnar}^{-/-}$ mice, also using Tubulin as a loading control.

Table S1 Core peptides predicted with high binding affinities to MHC molecules

Allele	Length (aa)	Predicted Peptide
H-2-Kb	8	ATYYFADL (Included in $VP1_{61\text{-}78}$ and $VP1_{67\text{-}84}$)
H2-IAb	12	PHQFINPRTNMT (Included in $VP2_{133\text{-}150}$ and $VP2_{139\text{-}156}$)
H-2-Db	9	GSIINNYYM (Included in $VP4_{13\text{-}30}$ and $VP4_{19\text{-}36}$)
